# Angiogenic Role of Mesothelium-Derived Chemokine CXCL1 During Unfavorable Peritoneal Tissue Remodeling in Patients Receiving Peritoneal Dialysis as Renal Replacement Therapy

**DOI:** 10.3389/fimmu.2022.821681

**Published:** 2022-02-04

**Authors:** Rusan Ali Catar, Maria Bartosova, Edyta Kawka, Lei Chen, Iva Marinovic, Conghui Zhang, Hongfan Zhao, Dashan Wu, Daniel Zickler, Honorata Stadnik, Marek Karczewski, Julian Kamhieh-Milz, Achim Jörres, Guido Moll, Claus Peter Schmitt, Janusz Witowski

**Affiliations:** ^1^ Department of Nephrology and Internal Intensive Care Medicine, Charité Universitätsmedizin Berlin, Corporate Member of Freie Universität Berlin and Humboldt-Universität zu Berlin, and Berlin Institute of Health (BIH), Berlin, Germany; ^2^ Division of Pediatric Nephrology, Centre for Pediatric and Adolescent Medicine, University of Heidelberg, Heidelberg, Germany; ^3^ Department of Pathophysiology, Poznan University of Medical Sciences, Poznan, Poland; ^4^ Department of General and Transplant Surgery, Poznan University of Medical Sciences, Poznan, Poland; ^5^ Institute of Transfusion Medicine, Charité Universitätsmedizin Berlin, Berlin, Germany; ^6^ Department of Medicine I, Nephrology, Transplantation and Medical Intensive Care, University Witten/Herdecke, Medical Centre Cologne-Merheim, Cologne, Germany; ^7^ Berlin Institute of Health (BIH) Center for Regenerative Therapies (BCRT), Charité Universitätsmedizin Berlin, Berlin, Germany; ^8^ Berlin-Brandenburg School for Regenerative Therapies (BSRT), Charité Universitätsmedizin Berlin, Berlin, Germany

**Keywords:** end-stage renal disease (ESRD), peritoneal dialysis (PD), mesothelium, cytokine/chemokine-signaling, angiogenesis, interleukin 17, CXC chemokine ligand 1 (CXCL1), COVID-19

## Abstract

Peritoneal dialysis (PD) is a valuable ‘home treatment’ option, even more so during the ongoing Coronavirus pandemic. However, the long-term use of PD is limited by unfavourable tissue remodelling in the peritoneal membrane, which is associated with inflammation-induced angiogenesis. This appears to be driven primarily through vascular endothelial growth factor (VEGF), while the involvement of other angiogenic signaling pathways is still poorly understood. Here, we have identified the crucial contribution of mesothelial cell-derived angiogenic CXC chemokine ligand 1 (CXCL1) to peritoneal angiogenesis in PD. CXCL1 expression and peritoneal microvessel density were analysed in biopsies obtained by the International Peritoneal Biobank (NCT01893710 at www.clinicaltrials.gov), comparing 13 children with end-stage kidney disease before initiating PD to 43 children on chronic PD. The angiogenic potential of mesothelial cell-derived CXCL1 was assessed *in vitro* by measuring endothelial tube formation of human microvascular endothelial cells (HMECs) treated with conditioned medium from human peritoneal mesothelial cells (HPMCs) stimulated to release CXCL1 by treatment with either recombinant IL-17 or PD effluent. We found that the capillary density in the human peritoneum correlated with local CXCL1 expression. Both CXCL1 expression and microvessel density were higher in PD patients than in the age-matched patients prior to initiation of PD. Exposure of HMECs to recombinant CXCL1 or conditioned medium from IL-17-stimulated HPMCs resulted in increased endothelial tube formation, while selective inhibition of mesothelial CXCL1 production by specific antibodies or through silencing of relevant transcription factors abolished the proangiogenic effect of HPMC-conditioned medium. In conclusion, peritoneal mesothelium-derived CXCL1 promotes endothelial tube formation *in vitro* and associates with peritoneal microvessel density in uremic patients undergoing PD, thus providing novel targets for therapeutic intervention to prolong PD therapy.

## Introduction

**Figure 1:**
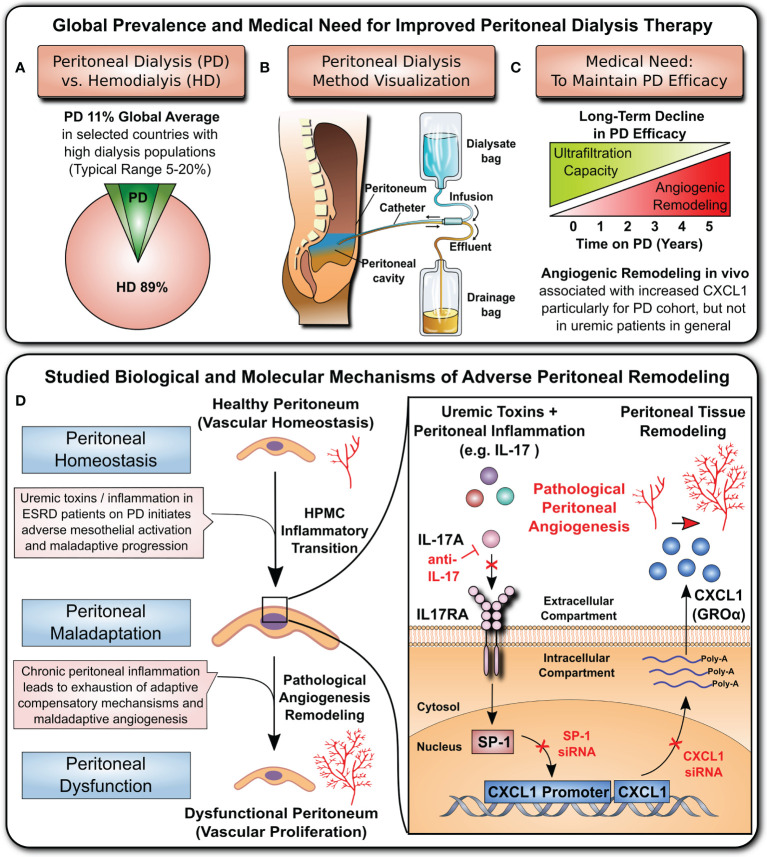
Clinical background and studied mechanism. **(A)** Global prevalence of hemodialysis (HD) and peritoneal dialysis (PD) (top left panel): hemodialysis is the most common method of RRT, while PD is thought to be underutilized by only ~11% of patients (8, 9). **(B)** Visualization of the PD principle (top central panel): Dialysis fluid is infused into the peritoneal cavity through a catheter. The fluid absorbs toxic waste products and excess water from blood vessels of the peritoneum and then is drained into the effluent bag. **(C)** Medical need (top right panel): The long-term efficacy of PD is hampered by loss of the ultrafiltration capacity of the peritoneal membrane due to detrimental remodelling and angiogenesis. **(D)** Cellular and molecular mechanism underlying peritoneal angiogenesis studied in this manuscript (lower panel): The cytokine interleukin 17 (IL-17) acts on peritoneal mesothelial cells and activates the nuclear transcription factor SP1, which leads to CXCL1 promoter activation, mRNA production, protein synthesis and release into the extracellular space. The released CXCL1 is a potent angiogenic stimulus, and the amount of CXCL1 in the peritoneal membrane correlates with the density of peritoneal microvessels.

Kidney disease is a major public health burden with growing medical need during the ongoing SARS-CoV-2 coronavirus pandemic (1–4). Patients with kidney failure requiring regular renal replacement therapy (RRT) are at particular risk of infection and Coronavirus-induced disease 2019 (COVID-19) (5–7). In addition to commonly used hemodialysis (HD) (**Figure 1A**), peritoneal dialysis (PD) (**Figure 1B**) is a valuable cost-effective ‘home-care’ RRT, which combines patients’ independence from HD centres with self-isolation, and thus is of particular interest during the COVID-19 pandemic (10–15).

The effective long-term use of the peritoneal membrane as a dialysis organ is still limited due to the inflammation-induced deterioration of peritoneal membrane function, resulting from peritoneal remodelling and angiogenesis (**Figure 1C**). Here, we have studied a novel CXC chemokine ligand 1 (CXCL1)-dependent angiogenic mechanism (**Figure 1D**) that leads to peritoneal angiogenesis independent of vascular endothelial growth factor (VEGF), which is typically associated with maladaptive angiogenesis in the RRT setting (8, 16–19).

Maladaptive angiogenesis in the peritoneal membrane contributes to ultrafiltration dysfunction and eventually leads to RRT failure (20–22). When using conventional PD fluids, the structural changes in the peritoneum include gradual thickening of the sub-mesothelial collagenous zone, and hyalinization and narrowing of blood vessels (23). In addition, patients with membrane failure usually have a markedly increased density of peritoneal blood vessels compared with patients with an uncomplicated course of PD (23, 24). The development of these alterations is presumed to be related to long-term exposure to bioincompatible dialysis fluid components (21). Intriguingly, a marked increase in peritoneal vascularity has already been observed shortly after initiation of PD in paediatric patients, when using solutions with a seemingly improved biocompatibility profile (25).

Other factors implicated in adverse peritoneal remodelling include repeated episodes of peritonitis and declining residual renal function (21). The development of detrimental peritoneal angiogenesis in PD increases the vascular area available for solute transport (21). This leads to increased absorption of glucose from PD fluids and premature loss of the glucose osmotic gradient necessary for ultrafiltration and fluid removal, thus impairing PD (26, 27). Indeed, the density of peritoneal microvessels predicts glucose transport independent of other factors (25). On the molecular level, VEGF is the best characterized mediator of angiogenesis (28) and it plays a role in peritoneal membrane dysfunction in PD (17, 29, 30). Prior studies identified the mesothelium as an important source of VEGF in the peritoneum and deciphered pathways of VEGF induction during PD (16, 17, 31–34). However, VEGF is not the only mediator of angiogenesis and prior attempts to inhibit VEGF in cancer revealed the existence of alternative VEGF-independent angiogenic programs, including one that can be initiated by IL-17 (35). Interestingly, CXCL1 exhibits proangiogenic activity that is related to presence of the three amino acid motif ELR (Glu-Leu-Arg) in its N-terminal domain (36–38). Proangiogenic activity of ELR^+^-CXC chemokines has been documented in tumorigenesis, corneal neovascularization, and pulmonary fibrosis (39, 40). CXCL1 is a potent neutrophil chemoattractant (41) that can be induced in human peritoneal mesothelial cells (HPMCs) upon stimulation with pro-inflammatory cytokines, such as TNFα, IL-1β and IL-17 (42–44). Mesothelial cell-derived CXCL1 plays the fundamental role in peritoneal host defence by recruiting neutrophils and enabling them to form elaborate traps for invading microorganisms on the mesothelial cells surface (45). However, little is known about whether CXCL1 can promote peritoneal angiogenesis during PD.

Recently, we have characterized the mechanism by which IL-17 induces CXCL1 in HPMCs and how this contributes to neutrophil recruitment during peritonitis (43, 46). Here, we have examined how IL-17-induced CXCL1 is involved in peritoneal angiogenesis in PD.

## Methods

### Patient Characteristics and Peritoneal Biopsy Collection

Peritoneal biopsies were collected by the International Peritoneal Biobank in accordance with the Declaration of Helsinki and according to the standardized protocol registered at www.clinicaltrials.gov (NCT01893710) (25). The patient characteristics are summarized in **Table 1**. For the current analysis, samples from 13 children with stage 5 chronic kidney disease (CKD5) and 43 children on chronic PD were selected. Groups were carefully matched by age to normalize for any changes in the density of peritoneal capillaries that may occur with age (47). Samples from CKD5 patients were collected at the time of the catheter insertion in preparation for PD. In the PD group, samples were obtained from children dialyzed with neutral-pH low-GDP fluids for no less than one month. The specimen was taken at a distance of at least 5 cm from the catheter exit site. Patients with a history of recent (<5 weeks) peritonitis were included only if successfully treated and fully recovered.

**Table 1:** Patient characteristics and functional parameters related to PD and CKD5.

Patient Parameter	CKD5 (n=13)	PD (n=43)	P-value
**Age (years)**	8.2 (1.8, 12.9)	6.0 (2.5, 12.2)	0.897
**PD duration (months)**	n.a.	15 (7, 32)	n.a.
**Glucose exposure (g/day/m^2^)**	n.a.	103 (68, 152)	n.a.
**Albumin (g/l)**	37.5 (33.2, 42.6)	34.5 (30.2, 38.6)	0.198
**Creatinine (mg/dl)**	5.2 (3.8, 7.6)	6.8 (4.2, 8.6)	0.265
**Hb (g/dl)**	11.0 (10.7, 12.1)	11.3 (10.0, 11.9)	0.853
**Ca (mmol/l)**	2.4 (2.2, 2.5)	2.4 (2.3, 2.5)	0.538
**P (mmol/l)**	1.9 (1.6, 2.1)	1.6 (1.3, 1.9)	0.131
**PTH (pmol/l)**	26 (19, 41)	23 (12, 49)	0.649
**BUN (mg/dl)**	44.8 (29.9, 70.0)	42.5 (27.0, 56.9)	0.432
**History of peritonitis (n, %)**	n.a.	12 (28%)	n.a.

Data presented as medians with interquartile range; CKD5, chronic kidney disease stage 5; PD, peritoneal dialysis; Hb, hemoglobin; Ca, calcium; P, phosphorus; PTH, parathyroid hormone; and BUN, blood urea nitrogen.

### Tissue Immunohistochemistry

Tissue staining was performed on formalin fixed, paraffin embedded 3 µm-thick tissue slices according to standard procedures. Following dewaxing, rehydrating, exposure to antigen retrieval buffer (Dako, Agilent, Santa Clara, CA, USA) and blocking of endogenous peroxidases (Dako REAL^®^, Agilent), primary antibodies in a background-reducing diluent (Agilent) were applied at 4°C overnight. The diluent with no antibody was used as a negative control. Appropriate biotinylated secondary antibodies were applied at room temperature for 30 minutes, followed by the avidin-biotin complex (Vector Labs, Burlingame, CA, USA) and 3′,3′-diaminobenzidine (DAB+, Agilent). Cell nuclei were counterstained with haematoxylin (Leica, Wetzlar, Germany). All antibodies are listed in **Table 2**.

**Table 2:** List of antibodies used in this study.

Antibody	Type/Clone	Source	Application	Dilution/Concentration
**CXCL1**	Rabbit polyclonal IgG	Abcam, Cambridge, UK; (#ab86436)	IHC	1:500
**CXCL1**	Polyclonal goat IgG	R&D Systems, Bio-Techne, Wiesbaden, Germany; (#BAF275)	Blocking	1 µg/mL
**Isotype IgG control for anti-CXCL1**	Polyclonal goat IgG	Thermo Fisher Scientific, Waltham, MA, USA; (#02-6202)	Blocking	1 µg/ml
**CD31**	Mouse monoclonal (clone JC70A) IgG	Agilent, Santa Clara, CA, USA; (#M0823)	IHC	1:25
**IL-17**	Mouse monoclonal (clone 4K5F6) IgG	Abcam, Cambridge, UK; (#ab189377)	IHC	1:50
**IL-17**	Mouse monoclonal (clone 41802) IgG	R&D Systems, Bio-Techne, Wiesbaden, Germany; (#MAB3171)	Blocking	0.5 µg/mL
**Isotype IgG control for anti-IL17**	Mouse monoclonal (clone #11711) IgG	R&D Systems, Bio-Techne, Wiesbaden, Germany; (#MAB002)	Blocking	0.5 µg/ml
**CD45**	Mouse monoclonal (clone 2B11 + PD7/26) IgG	Agilent, Santa Clara, CA, USA; (#M0701)	IHC	1:100
**Secondary anti-mouse**	Goat anti-mouse IgG	Vector Laboratories, Burlingame, CA, USA	IHC	1:100
**Secondary anti-rabbit**	Goat anti-rabbit IgG	Vector Laboratories, Burlingame, CA, USA	IHC	1:300

IHC, immunohistochemistry; IgG, immunoglobulin G; CD31/45, clusters of differentiation number 31 or 45; IL-17, interleukin 17; and CXCL1, CXC chemokine ligand 1.

Histological images were captured at 20× or 40× magnification (resolution 0.46 μm/pixel) using the Hamamatsu NanoZoomer 2.0-HT Scan System (Hamamatsu Photonics, Hamamatsu, Japan). All staining was digitally analysed using Aperio^®^ Precision Image Analysis Software and Image Scope version 11 (Aperio^®^ Technologies, Inc., Vista, CA, USA), applying algorithms, as described previously (47).

### Human Peritoneal Mesothelial Cell Culture

HPMCs were isolated from the specimens of omentum obtained from consenting patients undergoing elective abdominal surgery, as described elsewhere (16, 48). The cells were rendered quiescent by serum deprivation for 48 hours and then stimulated with recombinant cytokines (all from R&D Systems, Bio-Techne; Wiesbaden, Germany) or dialysate effluent, as specified in the legends to figures. All experiments were performed with cells no older than from the third passage to minimize the number of senescent cells.

### Endothelial Cell Culture and Tube Assay

Human microvascular endothelial cells (HMECs; catalogue no. CRL-3243) were purchased from ATCC^®^ (Manassas, VA, USA) and used at passages 2–6 (19). Endothelial tube formation assays were performed as introduced earlier (17, 19, 49). Briefly, Matrigel (Corning, Tewksbury, MA, USA) was poured into a 96-well plate (50 µl/well) and solidified at 37°C for 30 min. HMECs were seeded onto the Matrigel at density of 2×10^4^ cells/well (50) and cultured in MCDB131 medium (Thermo Fisher Scientific, Waltham, MA, USA) with or without 10% (v/v) conditioned medium from HPMCs pre-treated as described in the figure legends. Capillary networks of tubes formed were photographed under the microscope (Zeiss Axiovert 40 CFL Oberkochen, Germany) and five randomly selected fields from each well were analysed for the number of newly formed segments, junctions and meshes, using the Angiogenesis Analyzer on ImageJ 1.43 software (National Institutes of Health, Bethesda, MD, USA), as exemplified in **Figure 3B**.

### Gene Expression Analysis and Transfection Studies

Gene expression was assessed with reverse transcription quantitative PCR (RT-qPCR), as described earlier (16, 17, 19, 51). PCR primer sequences were as follows: β2-microglobulin (*β2M*; GenBank NM_004048.2) forward primer 5’-GTGCTCGCGCTACTCTCTCT-3’ and reverse primer (5’-CGGCAGGCATACTCATCTTT -3’), and *CXCL1* (GenBank NM_001511.4) forward primer 5’- AGGGAATTCACCCCAAGAAC-3’) and reverse primer 5’- TAACTATGGGGGATGCAGGA-3’. Transient transfection and luciferase assays were performed as previously described in detail (16, 19). Transfections with siRNAs were performed with the siRNA Transfection Reagent and siRNAs for *CXCL1* (sc-43816), *SP1* (sc-29487), or with scrambled siRNA control (sc-37007), as per manufacturer’s instructions (all materials were from Santa Cruz Biotechnology (Heidelberg, Germany).

### Immunoassays

The CXCL1 concentration in cell culture supernatants was measured using a DuoSet Immunoassay Kit (R&D Systems, Bio-Techne; Wiesbaden, Germany) (43, 44).

### Peritoneal Dialysis Effluent

Samples of peritoneal dialysis effluent (PDE) were collected and processed essentially as described previously (52). Cytokine concentrations in PDE were measured using Quantikine ELISA kits (R&D Systems, Wiesbaden, Germany). An exemplary infected PDE was collected from a patient presenting with an episode of acute *Enterobacter cloacae*-induced peritonitis. Cytokine concentrations in this PDE were as follows: IL-17 – 4 pg/ml, TNFα – 231 pg/ml, and CXCL1 - 471 pg/ml. In addition, PDE was collected from 3 stable patients on PD after a routine overnight PD. PDE from these patients did not contain detectable amounts of IL-17, IL-1β, and TNFα.

### Statistics

Statistical analysis was performed using GraphPad Prism 9.3.0 software (GraphPad Software, La Jolla, CA, USA). Data from *in vitro* experiments were analysed with ANOVA. Human samples were analysed the Mann-Whitney test, Fisher’s test and Spearman correlation. Results from *in vitro* experiments were expressed as the mean (± SD) fold change from the control. Other results are presented as specified in the legends to figures and tables. Findings with a P value <0.05 were considered significant. Asterisks represent P values as follows: * for P<0.05, ** for P<0.01, *** for P<0.001, and **** for P<0.0001.

## Results

### Clinical Background and Patient Description

In addition to the pre-existing high incidence of CKD and socioeconomic burden of RRT (1–3), the recent COVID-19 pandemic has led to a further increase in kidney disease cases, with 25% requiring RRT (4). Hemodialysis (HD) is the most common method of RRT (8, 9), while PD is used by ~11% of patients worldwide (with large regional differences ranging from 0% to 75%) (9) (**Figure 1A** **)**. Being more suited for ‘home dialysis’, PD may reduce the risk of virus transmission to susceptible patients. A visualization of the PD method is shown in **Figure 1B**. Briefly, a dialysis fluid is infused into the peritoneal cavity of the abdomen through an implanted catheter. The fluid absorbs toxic waste products and excess water from blood vessels in the peritoneum and then is drained into the effluent bag. The long-term efficacy of the PD process is considerably hampered by the adverse structural remodelling and angiogenesis in the peritoneal membrane (**Figure 1C**), which substantially compromises its ultrafiltration capacity. Here, we show that inflammatory mediators acting on the mesothelium upregulate CXCL1, which in turn can promote the maladaptive angiogenesis that considerably impairs long-term PD efficacy (**Figure 1D** **)**.

Detailed patient characteristics are summarized in **Table 1**. For the comparative *in vivo* analysis in this study, the samples from 13 children with stage 5 CKD (CKD5; samples collected at time of catheter insertion for PD) and 43 children on chronic PD (with a median PD duration of 15 months and with a median glucose exposure of 103 g/day/m^2^) were selected. Both groups were carefully matched by age to normalize for any changes in the density of peritoneal capillaries that may occur with age (median age 8.2 and 6.0 years, for CKD5 and PD patients, respectively, P=0.897) (47). There were no apparent differences in the levels of serum albumin, creatinine, hemoglobin (Hb), calcium (Ca), phosphorus (P), parathyroid hormone (PTH), and blood urea nitrogen (BUN) between the two groups.

### CXCL1 Expression Correlates With Peritoneal Vascularity in Biopsy Material

**Figure 2:**
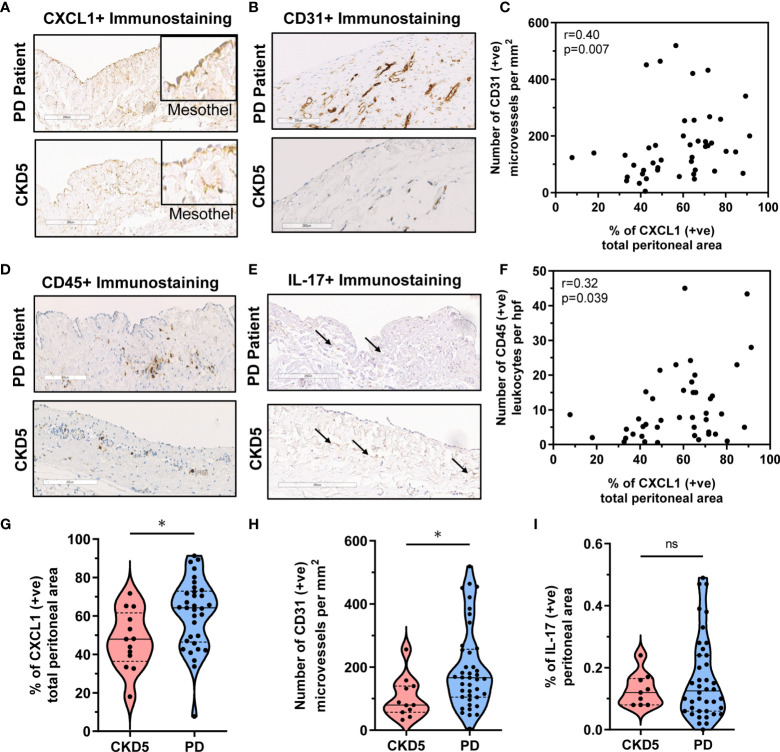
Correlation between peritoneal CXCL1 expression and CD31^+^ microvessels and CD45+ leukocyte infiltration in PD patients and CKD5 individuals. Peritoneal histology analysis comparing peritoneal dialysis (PD; n=43) patients and chronic kidney disease stage 5 (CKD5; n=13) patients before initiation of PD for expression of CXCL1, CD31, CD45 and IL-17: **(A, B)** Representative immunostaining for **(A)** CXCL1 (insets focusing on the mesothelium, magnification 20× or 40× in insets) and **(B)** CD31^+^ microvessels and **(C)** Spearman correlation between peritoneal CXCL1 staining and density of CD31-positive microvessels; and **(D, E)** Representative immunostaining for **(D)** CD45 and **(E)** IL-17 and **(F)** Spearman correlation between peritoneal CXCL1 staining and number of CD45-positive leukocytes; and **(G, H, I)** Violin plots with quantification of obtained results for CXCL1, CD31, and CD45, depicting the individual measurements, medians and quartiles.

First of all, we found that the peritoneum of both PD and CKD5 patients showed an extensive strong positive staining for CXCL1 (**Figure 2A** **)** with higher peritoneal CXCL1 expression in patients treated with PD, as quantified with observer-independent whole tissue digital image analysis (P<0.05, **Figure 2G**). This was evident in both mesothelial cells and submesothelial tissue. The difference in CXCL1 between PD and CKD5 patients was accompanied by a similar difference in the density of peritoneal microvessels (P<0.05, **Figures 2B, H** **)**. In an undivided group of PD and CKD5 patients, total peritoneal area staining for CXCL1 was associated with the density of CD31-positive microvessels (Spearman’s correlation r=0.40 and P=0.007, **Figure 2C**). This relationship was even stronger when expression of CXCL1 in the submesothelial tissue was tested (Spearman’s correlation r=0.618, n=45, P<0.0001, not shown). This may be due to the fact that in many specimens the surface mesothelium was not completely preserved (25). Samples with a discontinuous mesothelial monolayer were found more often in PD patients than in CKD5 patients (22/43 vs. 0/13, respectively, P=0.0007 obtained with Fisher’s test). In our earlier study we have occasionally observed incomplete mesothelial coverage of the peritoneum both in CKD5 patients and in healthy controls (25). In addition, peritoneal CXCL1 correlated with the number of CD45-positive leukocytes (Spearman’s correlation r=0.32, n=45, P<0.05, **Figures 2D, F** **)**, which were more abundant in PD patients than in CKD5 patients (P=0.0029; numbers per high power field (hpf) being 16 ± 12 vs. 4 ± 2, respectively). Only a weak staining for IL-17 (<0.5% positive area per peritoneal tissue area) could be detected in both PD and CKD5 patients (**Figure 2E**), with no major difference between the groups (ns, **Figure 2I**). In addition, in the analysed group of 43 PD patients, peritoneal expression of CXCL1 did not significantly correlate with the duration of therapy, the number of peritonitis episodes, or the total dialysis exposure to glucose (**Table 3**).

**Table 3:** Correlation between the abundance of peritoneal CXCL1 staining and selected clinical parameters in PD patients (n=43).

Parameter	Spearman’s rank correlation coefficient	P-value
Patients’ age (years)	-0.0959	0.5952 (ns)
PD duration (months)	0.1035	0.5666 (ns)
Total dialytic glucose exposure (g)	0.0190	0.9205 (ns)
Number of peritonitis episodes (n)	0.0982	0.5991 (ns)

ns, not significant.

### Mesothelial Cell-Conditioned Medium and Peritoneal Dialysate Promotes Endothelial Tube Formation by Microvascular HMECs in an IL-17 and CXCL1-Dependent Manner

To establish whether the observed association between CXCL1 expression and the density of peritoneal microvasculature in patients could be of a causal nature, we assessed the potential of CXCL1 to induce angiogenesis by using an *in vitro* endothelial tube formation assay, as employed in earlier studies by our group (17, 19, 49).

**Figure 3:**
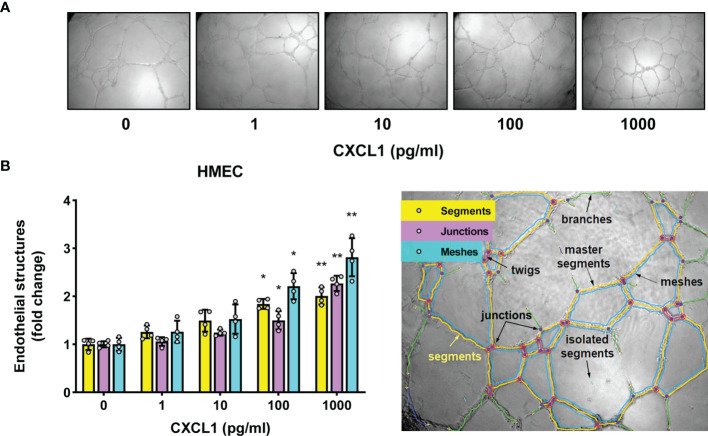
CXCL1 promotes tube formation by microvascular endothelial cells. **(A)** Representative microscopic images of HMECs (magnification 100x) embedded in Matrigel and treated for 16 hours with recombinant human CXCL1 at doses indicated; and **(B)** Quantification of the total segment length, and the number of junctions and meshes in CXCL1-treated HMECs (n=4, ANOVA) and an exemplary analysis of the parameters characterizing the endothelial network using the Angiogenesis Analyzer on ImageJ software. *P < 0.05 and **P < 0.01.

**Figure 4:**
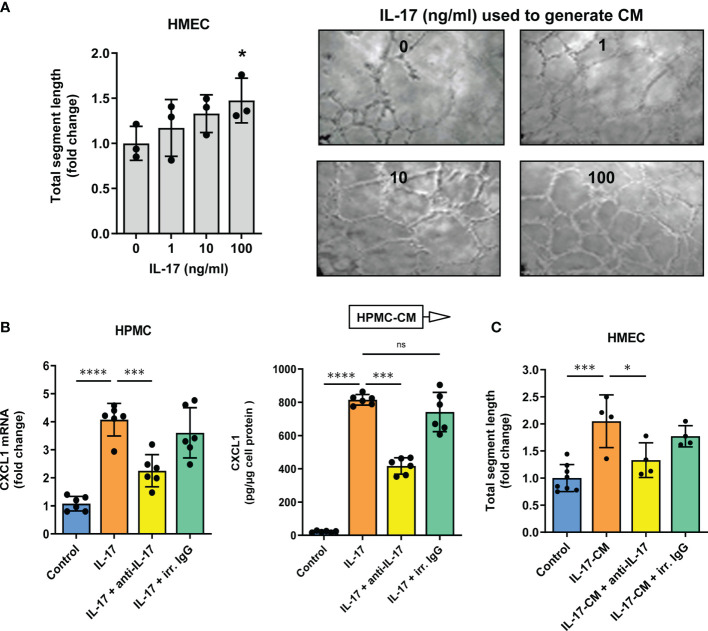
Conditioned medium from Il-17-stimulated mesothelial cells promotes tube formation by microvascular endothelial cells in an IL-17-dose dependent manner. Conditioned medium (CM) was collected from HPMCs treated with IL-17 for 24 hours and then added (10% v/v) to HMECs in Matrigel. After another 16 hours, the HMEC network was analysed; **(A)** Quantification of total segment length in HMECs exposed to CM from HPMCs stimulated with IL-17 at the indicated doses. In control, HMECs were incubated with CM derived from HPMCs not exposed to IL-17 (n=3, ANOVA). Representative images of thus treated HMECs are presented; **(B)** *CXCL1* mRNA levels (fold change) and cytokine levels (pg/µg of cell protein) in HPMCs stimulated with IL-17 (100 ng/ml) for 24 hours in the presence of either IL-17-blocking antibody or irrelevant (irr.) control antibody at the same dose of 1 µg/ml (n=6, ANOVA); and **(C)** CM generated as in *B* was added (10% v/v) to HMECs for 16 hours and total segment lengths were assessed. In control, HMECs were treated with CM from unstimulated HPMCs (n=4-8, ANOVA vs. cells exposed to CM from HPMCs treated with IL-17 alone). *P < 0.05, **P < 0.01, ***P < 0.001, ****P < 0.0001; ns, not significant.

Indeed, under these conditions recombinant CXCL1 was found to promote tubular endothelial morphogenesis in as dose-dependent manner (**Figures 3A, B** **)**. At the highest concentration of CXCL1 tested (1000 pg/ml), the total length of newly formed endothelial segments was almost twice as much as in untreated controls (P<0.01, **Figure 3B**). These segments were more connected to form junctions and meshes. The length of segments correlated strongly with both the number of junctions (Spearman r=0.86, p<0.0001) and the number of meshes (Spearman r=0.91, p<0.0001). Therefore the results of subsequent experiments were expressed as changes in the total segment length. Since we had previously observed that comparable amounts of CXCL1 could be secreted by HPMCs in response to IL-17 (43, 44), we next exposed HMECs to conditioned medium (CM) from HPMC stimulated with IL-17. Interestingly, the formation of endothelial tubes by HMECs treated in this manner increased in proportion to the dose of IL-17 used for HPMC stimulation (P<0.05, **Figure 4A**).

If, however, HPMC were stimulated with IL-17 in the presence of anti-IL-17 antibody, the strong upregulation of *CXCL1* mRNA expression and CXCL1 protein release by HPMCs (4-fold and 37-fold increase, respectively) was reduced by approximately 45% (P<0.0001 for both, **Figure 4B**) and the subsequent angiogenesis-promoting effect of CM was largely abolished (P<0.05, **Figure 4C**). Importantly, the use of a non-specific control antibody did not produce such an inhibition.

**Figure 5:**
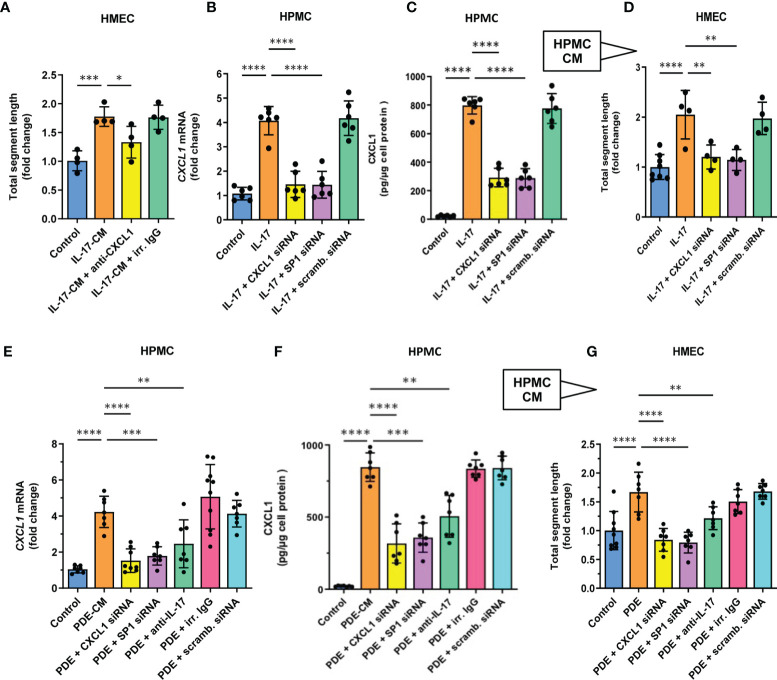
CXCL1 is a mediator of angiogenesis in mesothelial cell conditioned medium stimulated with either recombinant IL17 or IL-17-containing peritoneal dialysate. **(A)** HMECs were exposed for 16 hours to CM from IL-17-stimulated HPMCs in the presence of either CXCL1-neutralizing antibody or irrelevant (irr.) control IgG at the same dose (1 µg/ml), and analysed for total segment length. In control, HMECs were incubated with CM derived from HPMCs not exposed to IL-17 (n=4, ANOVA); and **(B, C)** HPMCs were transiently transfected with 10 nM *CXCL1* siRNA, *SP1* siRNA or scrambled (scramb.) control siRNA and then stimulated with IL-17 (100 ng/ml) for 24 hours and assessed for *CXCL1* mRNA or protein expression (fold change or pg/ug of protein, respectively; n=6, ANOVA); and **(D)** CM generated as generated in **(B, C)** was added (10% v/v) to HMECs for 16 hours and total segment lengths were assessed. In control, HMECs were treated with CM from unstimulated HPMCs (n=4-8, ANOVA); and **(E, F)** *CXCL1* mRNA and protein levels in HPMCs incubated for 24 hours with PD effluent (PDE, 25% v/v) from a patient with acute peritonitis. HPMCs were either transiently transfected with 10 nM siRNAs (*CXCL1*, *SP1*, scrambled control) or exposed to PDE in the presence of anti-IL-17 antibody or control IgG (1 µg/ml); and **(G)** CM generated as in **(E, F)** was added (10% v/v) to HMECs for 16 hours and total segment lengths were assessed. Data are presented as the mean (± SD) fold change from HMECs exposed to CM from HPMCs treated with PDE alone (n=4-8, ANOVA). *P < 0.05, **P < 0.01, ***P < 0.001, and ****P < 0.0001.

In order to determine whether the stimulatory effect of IL-17 was indeed mediated by CXCL1, the CM from IL-17-treated HPMCs was applied to HMECs together with CXCL1-neutralizing antibody (**Figure 5**). Indeed, the presence of anti-CXCL1, but not of control IgG, significantly reduced the stimulation of endothelial tube formation (P<0.05, **Figure 5A**). A similar inhibition of mesothelial CXCL1 induction occurred when the CM was collected from HPMCs treated with IL-17 in the presence of *CXCL1*-targeting siRNA, but not by the unspecific scrambled control siRNA. In this case, both the expression of *CXCL1* mRNA in HPMCs (P<0.0001, **Figure 5B**) and the release of CXCL1 protein by HPMC (P<0.0001, **Figure 5C**) and the subsequent degree of endothelial tube formation was significantly reduced (P<0.01, **Figure 5D**).

Since the production of CXCL1 by IL-17-stimulated HPMC was previously found to be controlled by the transcription factor SP1 (43), we also obtained the CM from HPMCs with *SP1* transiently silenced with the appropriate siRNA (**Figures 5B** **–D**). Similarly, these cells expressed significantly less *CXCL1* mRNA (P<0.0001, **Figure 5B**) and produced less CXCL1 protein (P<0.0001, **Figure 5C**) and the resultant angiogenic potential of their CM was also markedly reduced (P<0.01, **Figure 5D**).

CXCL1 can be induced in HPMCs *in vitro* not only in response to recombinant IL-17, but also to IL-17-containing peritoneal effluent from PD patients with peritonitis (43). Thus, we treated HPMCs with such a PD effluent (25% v/v) to generate CM (**Figures 5E** **–G**). When applied onto HPMCs, this PD effluent promoted a strong induction of *CXCL1* mRNA (P<0.0001, **Figure 5E**) and CXCL protein (P<0.0001, **Figure 5F**). Importantly, when the CM from these HPMCs was added to microvascular HMECs, it promoted a strong angiogenic response (P<0.0001, **Figure 5G**), and again this effect could be partially blocked when the anti-IL-17 antibody was added to PD effluent used to generate CM by HPMCs (P<0.01, **Figure 5G**).

Moreover, when the process of medium conditioning was performed on HPMCs treated with *CXCL1*-siRNA or *SP1*-siRNA, but not with the corresponding scrambled control siRNA, both CXCL1 mRNA induction and protein secretion, as well as endothelial tube formation-promoting activity of the CM was reduced to control levels (P<0.0001 and P<0.01, respectively, **Figures 5E** **–G**).

**Figure 6:**
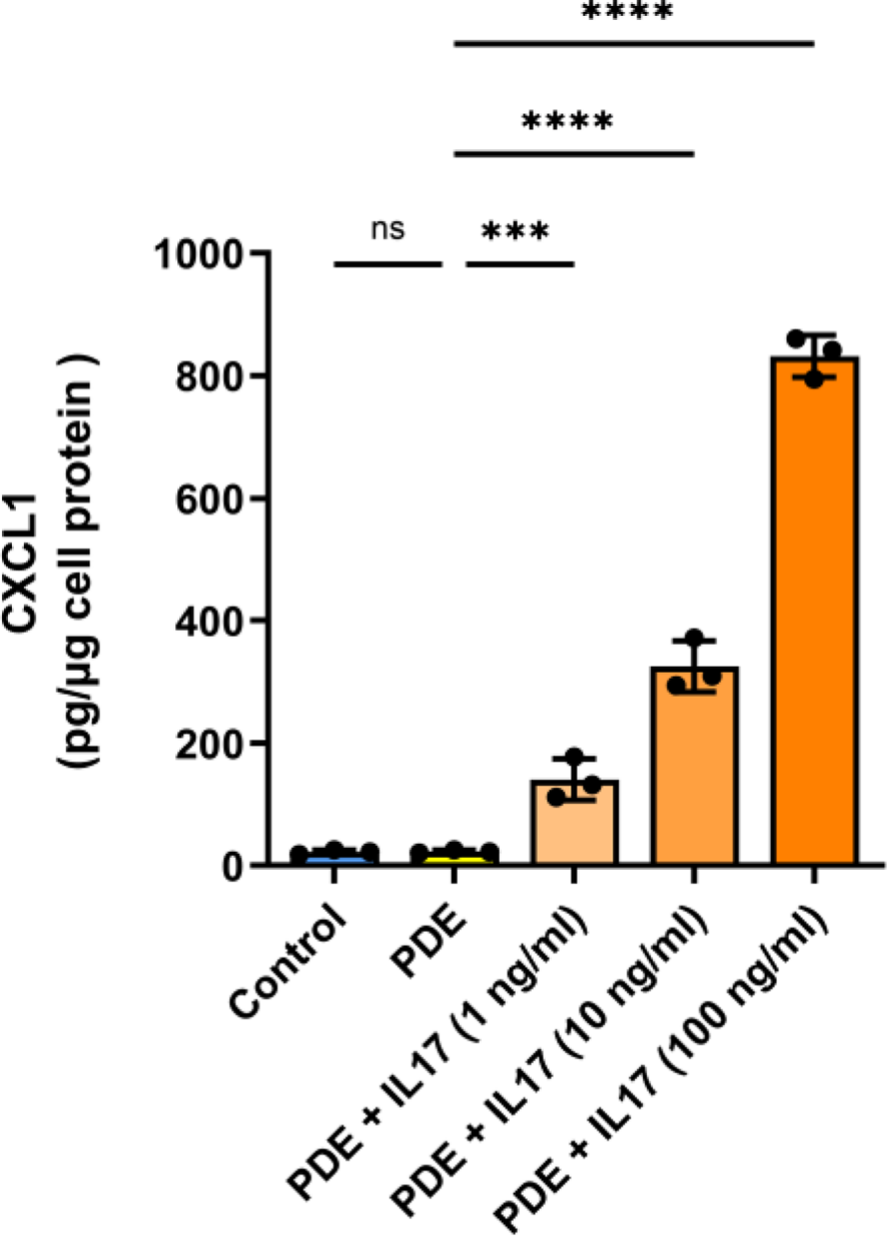
Spiking of recombinant IL-17 into peritoneal dialysis effluent promotes CXCL1 induction in a dose-dependent manner. PDEs from stable non-infected PD patients (n=3) were spiked with increasing doses of IL-17 (1, 10, 100 ng/ml) and applied to HPMC cultures with readout of CXCL1 protein production (pg/µg of protein) 24 hours later. ***P < 0.001, ****P < 0.0001; ns, not significant.

Next, we studied the potential consequences of elevated IL-17 presence in the (inflamed) peritoneum. The levels of IL-17 in peritoneal dialysis effluent (PDE) follow temporary peaks in patients with acute peritonitis (53, 54). However, these are rare events with approximately 0.5 episodes per patient-year (55), so the experiments shown in **Figures 5E** **–G** were performed with PDE from a single donor with high IL-17 levels. On the other hand, IL-17 concentrations often fall below the detection limit in PDE from stable donors without ongoing infection. Indeed, when studying PDE from non-infected PD patients we did not find any detectable amounts of IL-17 (data not shown) and such fluids did not promote CXCL1 secretion when added to HPMCs in culture (**Figure 6**). However, when these PDEs were spiked with increasing doses of IL-17, they began to stimulate CXCL1 secretion by HPMCs in an IL-17 dose-dependent manner, thus confirming the importance of IL-17 for CXCL1 induction in the peritoneal milieu established in the earlier experiments.

## Discussion

Kidney disease is now recognized as a major public health burden (1–3). The population prevalence of CKD is ~10% (1). The number of patients with CKD requiring RRT is rising steadily and projected to reach 5.4 million by 2030 (2). The situation has been aggravated by the recent SARS-CoV-2/COVID-19 pandemic, which has led to an increase in new cases of kidney disease (3), almost 25% of whom will require RRT (4). Thus, increased scientific efforts are currently ongoing to improve already existing well-established RRT methods, such as HD and PD, and to develop novel technologies (e.g. cellular therapy) to maintain, improve, or replace (lost) kidney (transplant) function (8, 56–58).

The socioeconomic burden of RRT is high, since conventional HD is rather costly for healthcare providers, with substantial differences in funds available per capita across different countries (58). As alternative to HD (8), PD is a well-established and viable option for home RRT, which minimizes potential virus exposure and the associated health risks (10–15). However, its long-term use is limited due to the decline in peritoneal ultrafiltration that typically occurs 2-4 years after initiation of PD (22).

Surprisingly, even PD using dialysis fluids regarded as biocompatible seems to induce early alterations and angiogenesis in the peritoneal membrane (25), which may initiate or accelerate the deterioration in peritoneal function. Such observations underline the need for more detailed studies of the angiogenesis pathways.

While it is recognized that the gradual decline in peritoneal membrane function during PD is partly due to adverse tissue remodeling and angiogenesis (21), the molecular mechanisms underlying these processes are still poorly understood to date (59). So far, vascular changes in the dialyzed peritoneum have been analysed almost solely in relation to VEGF expression (17). Although the role of VEGF in peritoneal angiogenesis is supported by the relationship between VEGF levels and solute transport rates (25, 60, 61), the involvement of other angiogenic factors has not yet been investigated in detail. The present study demonstrates the angiogenic potential of the mesothelium-derived chemokine CXCL1 *in vitro* and reveals its association with the density of peritoneal vasculature in patients on PD.

ELR^+^-CXC chemokines, as exemplified by CXCL1, have shown their potent angiogenic activity in contexts other than PD (36, 37, 39, 40) either by directly stimulating endothelial cells or by recruiting leukocytes, which then release other angiogenic stimuli, including VEGF (62). Here, we show that CXCL1 secreted by HPMCs can directly promote endothelial tube formation of microvascular HMECs. The mechanisms by which CXCL1 acts on endothelial cells are initiated by signalling through CXCR2 receptor (39) and include reorganization of cytoskeleton, activation of extracellular signal-regulated protein kinases, enhanced cell proliferation, and migration (63).

Secreted CXCL1 is largely immobilized on cell surfaces or extracellular matrix, including mesothelial cells and the peritoneum (43, 45). Indeed, in the peritoneal biopsy analysis we have observed an extensive presence of CXCL1 in both the mesothelium and the submesothelial tissue. Importantly, by interacting with various glycosaminoglycans (64), CXCL1 can be retained in select tissue compartments, which facilitates the formation of gradients guiding the migration of CXCR2-expressing leukocytes or endothelial cells.

In addition, CXCL1 is possibly produced more extensively by HPMCs that have undergone epithelial-to-mesenchymal transition (EMT) and have migrated from the peritoneal surface into the interstitium (65). This would explain the abundant CXCL1 staining even in samples without the preserved mesothelial monolayer, as shown in the present study. Interestingly, it has been observed previously that PD patients with evidence of EMT had more microvessels in the submesothelial area compared to patients without EMT (25). Also cells other than mesothelial cells, such as peritoneal fibroblasts (52), macrophages (66, 67), and endothelial cells (68), can contribute to the peritoneal production of CXCL1.

Both ELR^+^-CXC chemokines and VEGF can interact during angiogenesis by triggering each other’s expression or by modulating the signalling from their receptors (62). The extent of these interactions is further modulated by the local specificity of endothelial cells involved and the underlying pathological condition. Additional level of regulation may be related to the type of stimulus for angiogenic factor production. In this respect, IL-17 has drawn attention as a trigger for mobilization and recruitment of myeloid cells capable of secreting angiogenic factors independent of VEGF (35). To study the interplay between of VEGF and other angiogenic factors and the exact involvement of IL-17 requires further in-depth *in vitro* studies and validation by *ex-vivo* analysis of human peritoneal tissue.

The importance of IL-17 for intraperitoneal homeostasis in PD is increasingly recognized (46). While IL-17-producing T_H_17 and γδ T cells are found in the peritoneum only sporadically in healthy individuals, they seem to gradually accumulate in the dialyzed peritoneum, which correlates with inflammation and fibrosis (69). It has been demonstrated that differentiation of naïve T-cells into T_H_17 cells can be a consequence of PD fluid-induced osmotic and oxidative stress (70).

In this study, we focused on the effect of IL-17 on mesothelial cells, as they are one of the main producers of peritoneal CXCL1, as also shown in our patient peritoneal biopsy analysis. Although IL-17 can act directly on endothelial cells to stimulate CXCL1 (71), the contribution of this pathway is probably of lesser significance given that the conditioned medium from IL-17-stimulated mesothelial cells with silenced *CXCL1* gene expression did not promote endothelial angiogenesis. This observation indicates that IL-17 present in the conditioned medium had a negligible effect on the endothelium.

In the future, it may be of particular interest to investigate how peritoneal expression of CXCL1 changes with time on PD, as compared to changes in microvascular density and specific dialysis parameters. This may require longitudinal studies both in children and adults, as the immune response to PD and CKD may change with age. The tissues in children are uniquely suited to study CKD- and PD-induced peritoneal alterations, since they are hardly affected by lifestyle- and aging-related factors, and the diseases requiring RRT (such as congenital abnormalities of the kidney and urinary tract) do not *per se* affect the peritoneum.

Although the density of peritoneal blood capillaries changes with age in a U-shaped fashion (47), age alone did not predict peritoneal vessel density in multivariate analysis in children on PD (25). Still, in the smaller cohort of patients studied here, we can not rule out that age-related differences in vascular density may have masked the effects related to PD exposure. This may partially explain the lack of a clear correlation in our cross-sectional analysis between the time spent on PD and CXCL1 expression.

Due to the apparent toxicity of GDPs, fluids low in GDPs are increasingly used in clinical practice in most European dialysis centres (25). All children analysed in the present study were treated with such solutions. However, it has recently been demonstrated that even fluids with neutral pH and low GDP can induce substantial peritoneal angiogenesis, together with cellular infiltration, cytokine release and EMT (25). Thus, the role of CXCL1 could be of particular relevance in patients treated with such fluids.

In contrast, in children dialyzed with fluids with a high GDP content, angiogenesis and inflammatory cell infiltration is less pronounced, but associated with a diminished immune response, activation of cell death pathways and fibrosis, and accelerated arteriolopathy with significant lumen narrowing (72). Likewise, rapidly progressing peritoneal hyalinizing vasculopathy with no consistent increase in the number of CD31-positive vessels has been reported in adult patients treated with high GDP-containing fluids (73, 74). Thus, it becomes clear that further research in this direction is essential to understand this phenomenon.

## Conclusions and Limitations

We here show that CXCL1 induced by IL-17 in mesothelial cells displays angiogenic activity, which further adds to the complexity of mechanisms controlling vascular remodelling in the dialyzed peritoneum. This aspect of peritoneal angiogenesis control should be anticipated and understood in order to mitigate the adverse consequences of increased vascularity for PD. The main finding of this study is the apparent association between the extent of peritoneal CXCL1-positive staining and peritoneal vascularity and, secondly, the observation that CXCL1 expression and the density of microvessels increase in PD as compared to patients with CKD5, pointing to a PD-specific aspect of the CXCL1-induced peritoneal angiogenesis. Furthermore, we verified the relationship between observations in patients by detailed studies of the corresponding mechanisms studied *in vitro*. Based on our prior studies (43), we have chosen to employ IL-17 as one representative inducer of CXCL1 in the PD setting. However, also other factors, such as TNFα and IL-6, can have a triggering or amplifying effect on endothelial dysfunction in the context of (hemo)-dialysis (8). While the *in vitro* induction and blocking experiments using the peritonitis effluent clearly demonstrate the pro-angiogenic potential of the IL-17/CXCL1 axis, the histological material from a rather small group of PD patients showed no apparent association between peritoneal IL-17 expression and vascularity. However, in a previous study on a larger cohort of PD patients treated with both low- and high-GDP fluids, the abundance of peritoneal IL-17 indeed correlated with microvessel density (72), thus supporting our current results. As PD-associated peritoneal remodeling develops gradually over the years, we are now conducting additional studies in a larger group of patients to characterize the time course of CXCL1- and VEGF-induced peritoneal angiogenesis, relative to inflammatory pathways. These findings should provide an interesting starting point for future interventions aiming at preserving long-term peritoneal membrane integrity and function.

## Data Availability Statement

The raw data supporting the conclusions of this article will be made available by the authors, without undue reservation.

## Ethics Statement

The studies involving human participants were reviewed and approved by the International Peritoneal Biobank. Written informed consent to participate in this study was provided by the participants’ legal guardian/next of kin.

## Author Contributions

Study design and supervision: RC, MB, GM, CS, and JW. Sample collection, data acquisition, experimental work: RC, MB, EK, LC, IM, CZ, HZ, DW, DZ, HS, MK, and GM. Data analysis, interpretation and visualization: RC, MB, JK-M, AJ, GM, CS, and JW. Manuscript writing, review and editing: RC, MB, AJ, GM, CS, and JW. All authors contributed to the article and approved the submitted version.

## Funding

JW and EK were supported by the grant from the Narodowe Centrum Nauki (NCN, Polish National Science Centre; #2016/23/B/NZ4/03711). Contributions of RC were made possible by funding from the Deutsche Forschungsgemeinschaft (DFG, German Research Foundation; project #394046635, subproject A03) as part of CRC 1365. MB was funded by the DFG grant #419826430. CPS obtained funding from the European Union’s Horizon 2020 Research and Innovation Programme under the Marie Sklodowska-Curie grant agreement (#812699) and from the European Nephrology and Dialysis Institute (ENDI). GM’s contributions were made possible by funding from the German Federal Ministry for Education and Research (BMBF) and German Research Foundation (DFG) through the Berlin Institute of Healthy (BIH)-Center for Regenerative Therapies (BCRT) and the Berlin-Brandenburg School for Regenerative Therapies (BSRT, GSC203), respectively, and in part by the European Union’s Horizon 2020 Research and Innovation Program under grant agreements No 733006 (PACE) and 779293 (HIPGEN). We acknowledge support from the DFG and the Open Access Publication Fund of Charité – Universitätsmedizin Berlin.

## Conflict of Interest

The authors declare that the research was conducted in the absence of any commercial or financial relationships that could be construed as a potential conflict of interest.

## Publisher’s Note

All claims expressed in this article are solely those of the authors and do not necessarily represent those of their affiliated organizations, or those of the publisher, the editors and the reviewers. Any product that may be evaluated in this article, or claim that may be made by its manufacturer, is not guaranteed or endorsed by the publisher.
